# Characterizing, mapping and valuing the demand for forest recreation using crowdsourced social media data

**DOI:** 10.1371/journal.pone.0272406

**Published:** 2022-08-11

**Authors:** Federico Lingua, Nicholas C. Coops, Valentine Lafond, Christopher Gaston, Verena C. Griess

**Affiliations:** 1 Department of Forest Resources Management, Faculty of Forestry, Forest Sciences Centre, University of British Columbia, Vancouver, BC, Canada; 2 Department of Wood Science, Faculty of Forestry, Forest Sciences Centre, University of British Columbia, Vancouver, BC, Canada; 3 Institute of Terrestrial Ecosystems, Department of Environmental System Sciences, ETH Zurich, Zurich, Switzerland; Zoological Survey of India, INDIA

## Abstract

Mapping and valuing of forest recreation is time-consuming and complex, hampering its inclusion in forest management plans and hence the achievement of a fully sustainable forest management. In this study, we explore the potential of crowdsourced social media data in tackling the mapping and valuing of forest recreation demand. To do so, we assess the relationships between crowdsourced social media data, acquired from over 350,000 Flickr geotagged pictures, and demand for forest recreation in British Columbia (BC) forests. We first identify temporal and spatial trends of forest recreation demand, as well as the countries of origin of BC forests visitors. Second, we estimate the average number of annual recreational visits with a linear regression model calibrated with empirically collected secondary data. Lastly, we estimate recreational values by deriving the average consumer surpluses for the visitors of BC forested provincial parks. We find that annually, on average, over 44 million recreational experiences are completed in BC forests, with peaks during the summer months and during the weekends. Moreover, a crowdsourced travel cost approach allowed us to value the recreational ecosystem service in five forested provincial parks ranging from ~2.9 to ~35.0 million CAN$/year. Our findings demonstrate that social media data can be used to characterize, quantify and map the demand for forest recreation (especially in peri-urban forests), representing a useful tool for the inclusion of recreational values in forest management. Finally, we address the limitations of crowdsourced social media data in the study of forest recreation and the future perspectives of this rapidly growing research field.

## Introduction

Rapid concentrations of populations into urban areas [1] are placing significant pressure on the surrounding ecosystems. Recreational activities in forested ecosystems reachable from urban centers have shown the potential to mitigate a number of urban health concerns, such as lack of exercise and obesity [2], as well as mental health problems [3]. For this reason, recreation plays a role of growing importance in the array of ecosystem services that forests provide to humans [4].Therefore, providing access to forest recreational opportunities is of increasing importance on political agendas worldwide [5]. Even though the perceived importance and the number of people engaging in leisure activities in forest ecosystems are increasing globally [6], including recreational values in forest management plans is still challenging [7] and entails difficulties with both mapping [8] and valuing [9] these services. Challenges in mapping forest recreation (and cultural ecosystem services in general) mainly originate from the lack of available fine scale, spatially explicit data [10]. Challenges in valuing forest recreation (and non-market ecosystem services in general) arise from the lack of market price [11]. Approaches available to attribute a monetary value to forest recreation can be grouped in two different categories: (i) the stated preference approach, which relies on hypothetical market settings, and (ii) the revealed preference approach, which relies on actual decisions that people make. Revealed preference approaches, such as the travel cost method and hedonic pricing, are generally considered the best options [12]. However, the data collection phase of revealed preference methods is laborious and time-consuming, limiting the number of their applications [13]. Despite the inherent difficulties in mapping and valuing forest recreation, managing an ecosystem without considering the entire range of services it provides may lead to its depletion and ultimately jeopardize the sustainability of humans on planet Earth [14].

Fortunately, during the last decade, a new data source for the study of nature-based recreation has become available: passive crowdsourced social media data. Social media data (such as texts, pictures and videos) are being used in a rapidly growing number of studies [15] throughout various environmental disciplines [16]. For example, counts of geotagged pictures can be used to infer the number of visits to a natural landscape and hence to map the consumption of forest recreation at large scale, with high temporal and spatial resolution. In fact, geotagged picture counts have shown strong correlation, both spatially and temporally, with empirically determined numbers of visits across various natural ecosystems [17–19]. Studies that infer the number of visits from geotagged picture counts adopt regression models calibrated on empirically collected data. Most of these studies dealt with the recreational ecosystem provided by either fresh water bodies [20, 21], mountain landscapes [22]. More recently, Wood et al. [23] used linear fixed effects models to estimate the number of visitors in unmonitored recreational sites, using weather condition, time of year and number of social media posts as independent variables. Crowdsourced social media data can also be successfully used to infer the place of origin of people recreating in the natural landscape, enabling the segmentation of visitors into domestic and international [24]. Lastly, recent studies conducted on wetlands [21] and National Parks [25] have shown that crowdsourced social media data can be effective in informing traditional revealed preference valuation methods for non-market values (specifically the travel cost method).

Despite the rapidly growing number of studies that use crowdsourced social media data in environmental research, this discipline is still in its infancy [16]. Managed forests have so far been the subject of a very limited number of such studies. To the best of our knowledge [26–28], are the only three studies that used crowdsourced social media data to explore the recreation ecosystem service provided by managed forests. The first two studies focused on identifying variables (stands composition, topography, etc.) that positively influence the number of pictures acquired by visitors, while [28] explored the potential that social media data have in assessing visitor frequencies in urban and peri-urban forests. To our knowledge, crowdsourced social media data has never been used before for travel cost method applications conducted in forest recreational sites.

Given the importance of including recreation in forest policies and forest management decisions, and the opportunities that crowdsourced social media data could offer to this end, in this study we use Flickr geotagged pictures to characterize, map, and value the recreation ecosystem services provided by British Columbia’s (BC) forests, Canada. BC forests are a highly relevant example of an ecosystem that provides diverse and often conflicting ecosystem services [29]. Timber provision and forest recreation have shown a clear trade-off relationship throughout the province however, both are instrumental for the economy [30]. The overarching objective of our study is to evaluate the potential of crowdsourced social media data in characterizing forest recreation and overcoming the mapping and valuing issues that have historically limited the consideration of cultural ecosystem services in forest management planning. We will structure our analysis on three levels. First, we will use Flickr geotagged pictures to characterize demand for recreation in BC forests, identifying temporal and geographic trends, as well as the place of origin of the recreationists. Second, we will map forest recreation in BC, assessing the average annual number of visits in BC forests from geotagged pictures. Third, we will value the recreational ecosystem service provided by BC forested provincial parks, estimating travel cost models from crowdsourced data. Lastly, in the light of the obtained results, we will discuss the opportunities and the limitations of this innovative data source in the study of forest recreation and its inclusion in forest management plans.

## Study area, data and methods

### Study area

**Fig 1:**
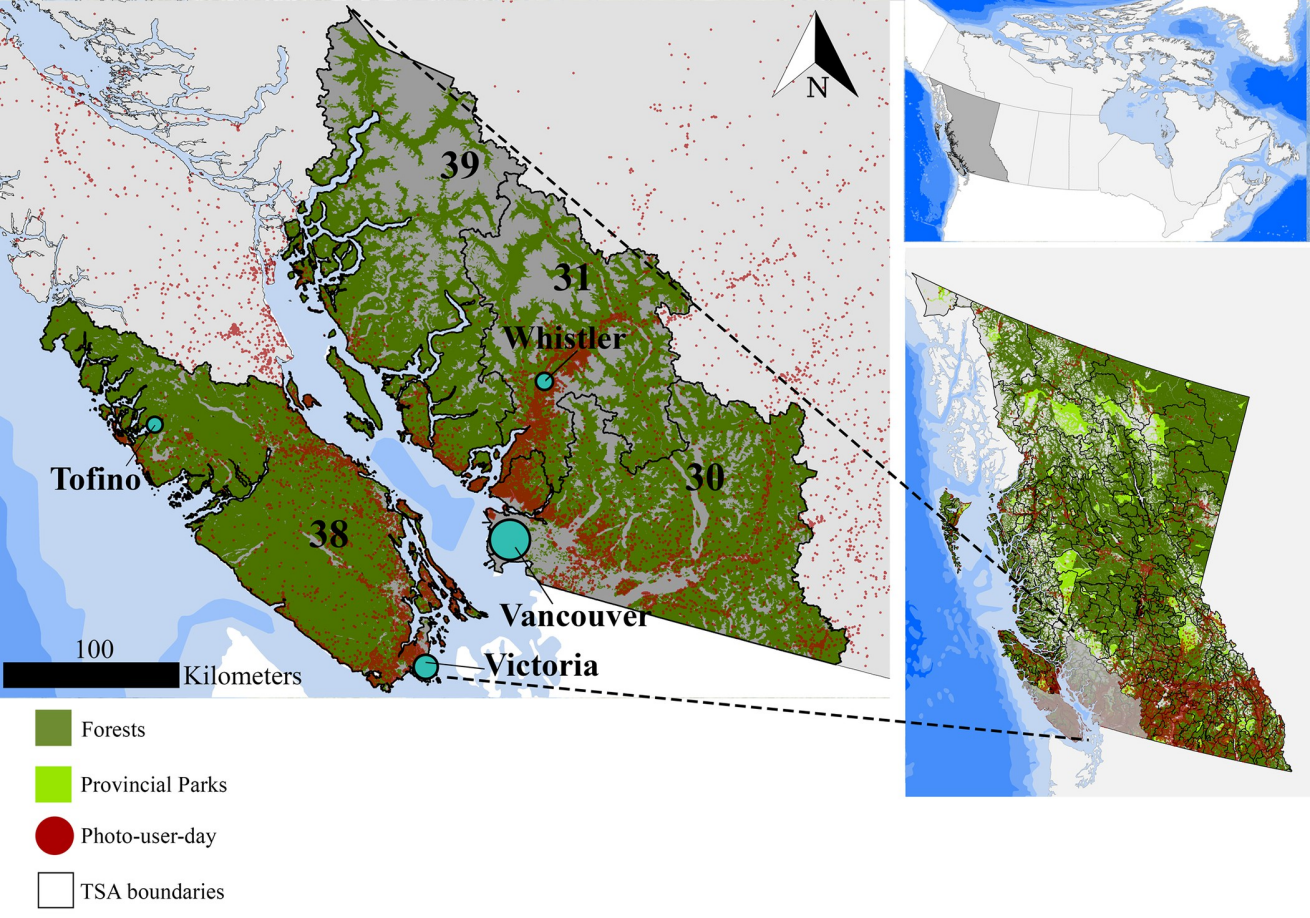
Description of the study area in BC. Forest cover (dark green), provincial parks (light green), Timber Supply areas (TSAs in black) and the location of single visits (photo-user-day, PUD) derived from crowdsourced social media data (red). Geodetic datum: NAD 83. Source of layers Natural Earth (https://www.naturalearthdata.com/).

The Province of BC is the westernmost province of Canada (Fig 1). BC is dominated by forested ecosystems, which cover 57% of the land area and are 95% publicly owned [31]. From an economic perspective, timber provisioning has traditionally been the main ecosystem service provided by BC forests. Timber supply areas (TSA) are located throughout the province based on the wood flow patterns from the management units to the main timber-using industries. Although we collected Flickr geotagged pictures and conducted the analysis for the entire province, to better visualize our results we will focus on south-western BC and use TSA 30, TSA 31, TSA 38, and TSA 39 as a relevant subset (Fig 1). The above-mentioned TSAs surround Vancouver and Victoria, the two largest population centers in BC.

### Data

As shown in Table 1, the data used in this study comprised three categories: (i) crowdsourced, (ii) geographic, and (iii) secondary (data previously collected through primary sources and made available for research). We developed a Python application and extracted from Flickr Application Programming Interface (API) the metadata of all the geotagged pictures acquired in BC between January 2005 and December 2020. Flickr is the most widely used social media website in the study of nature-based recreation [16] because of its easily accessible API. Moreover, Flickr was launched in 2004, offering the longest series of data among the photo-sharing social media platforms. Data were collected following terms and conditions of Flickr’s API. Furthermore, only the pictures uploaded by users that authorized their photos to be accessed by API-based searches done outside of the Flickr website were accessed and included in the analysis. Lastly, all the metadata were anonymized and analyzed in aggregate form.

**Table 1:** Data used to estimate the seasonal trends, number of annual visits of British Columbia’s (BC) forests and the value of the recreational ecosystem service. PUD = photo-user-days, API = Application Programming Interface.

Description	Typology	Source
PUDs in BC provincial parks for each Flickr user	Crowdsourced data	Flickr API
PUDs from geotagged pictures taken in BC	Crowdsourced Data	Flickr API
BC provincial park boundaries	Geographic data	BC data catalogue
BC forested area	Geographic data	BC data catalogue
Open Street Map Road Network	Geographic data	https://planet.openstreetmap.org/
BC wooded land boundaries	Geographic Data	BC data catalogue
BC park boundaries	Geographic Data	BC data catalogue
Grid with 10 km^2 cells	Geographic Data	ArcMap elaboration
Individual hourly income	Secondary data	BC data catalogue
Average cost of driving a private car (CAN$/km)	Secondary data	https://carcosts.caa.ca
Visitation data for BC parks	Secondary Data	BC Parks end of year reports
Global Human Influence Index	Secondary Data	SEDAC (NASA)

Retrieved metadata included: (i) unique identifier code for the picture, (ii) unique identifier code for the author of the picture, (iii) the date on which the picture was taken, (iv) the coordinates of where the picture was taken. Combining these metadata, we derived the Photo-User-Days (PUDs) in BC from 2005 to 2020, which are unique combinations of author and day used to account for the fact that a single social media user can acquire and upload multiple pictures during the same recreational experience [17]. For each PUD, a reference coordinate was then assigned as the geographic center (mean) of all grouped pictures.

Geographic data consisted of shapefiles defining: (i) the administrative boundaries of BC, (ii) the land cover map of BC, (iii) the boundaries of BC provincial parks, and (iv) the BC road network downloaded from Open Street Map using the Python package OSMnx. A BC land cover map was used to extract a forest mask to separate: relevant pictures (acquired in forested areas) and non-relevant pictures (acquired outside forested areas). Only the metadata corresponding to the relevant pictures were retained for further analyses. Lastly, using the tessellation tool in ArcMap we created a grid of 10 km^2^ cells covering the entire study area that was used to create the map of the estimated number of annual visits in BC forests.

Secondary data used in this study were: (i) average costs of driving a private car in BC [32], (ii) the estimated hourly income rate from BC census data [33], (iii) visitation statistics for BC parks collected by the BC Ministry of Environment and Climate Change [34], and (vi) global human influence index (GHII) [35]. GHII is a global dataset of 1-kilometer grid cells, created from different layers (accounting for human population pressure, land use, infrastructure, and human access) that provides an updated map of anthropogenic impacts useful for human-environment interactions research.

### Methods

The methodology that we designed to explore, map and value the recreational ecosystem service provided by BC forests from crowdsourced social media data can be subdivided in three steps. First, we used the crowdsourced geotagged pictures to infer a home location for the visitors of BC forests, as well as to identify temporal and geographic trends of recreational visits. Second, we created a model to assess the number of annual visits in BC forests based on the number of pictures extracted from Flickr. Third, we estimated recreational values in BC by calculating the average consumer surplus for various forested provincial parks combining pictures’ metadata with secondary data.

#### Visitors home location and visitation trends

In many of the previous studies that have inferred social media users’ home locations, locations were determined by extracting the information directly from each user’s public profile. However, as previously found by Da Rugna et al. [36], only 40–48% of Flickr users share a home location in their profile. In addition, shared home location may not be up to date. To overcome these issues, we estimated home locations using the max photo-user-days (max PUDs) method proposed by Bojic et al. [37] and verified by Sinclair et al. [24]. The max PUDs method assumes that the user’s home location can be estimated as the one in which they have spent the maximum number of PUDs. This methodology was applied to estimate the home location of each user with at least 10 geotagged pictures shared, while users with less pictures were excluded from further analyses. Retained visitors were divided in: (i) domestic (users having most PUDs within BC), and (ii) international (users having most PUDs outside BC borders). For domestic visitors, the max PUDs method was applied to infer the regional district of origin. We then estimated the coordinates of the home location of each domestic visitor as the geographic center (mean) of the pictures taken within their regional district of origin. To test the precision of the estimated home locations, we compared the results of the max PUDs method with the available countries of origin declared by users in their profiles. To identify BC hotspots of forest recreation consumption, we applied the optimized hotspot analysis tool in ArcGIS, using the geotags of the retrieved pictures. This tool divides the study area in cells of equal area and then calculates for each cell the probability of it being a hotspot, using the Getis-Ord Gi* statistic [38]. The cells area used by the optimized hotspot analysis tool was 5.4 km^2^, calculated by multiplying the median nearest neighbor distance by two, and then using this value to construct a hexagon polygon grid. To analyze temporal trends, we grouped pictures using their timestamps.

#### Annual visits in BC forests

**Fig 2:**
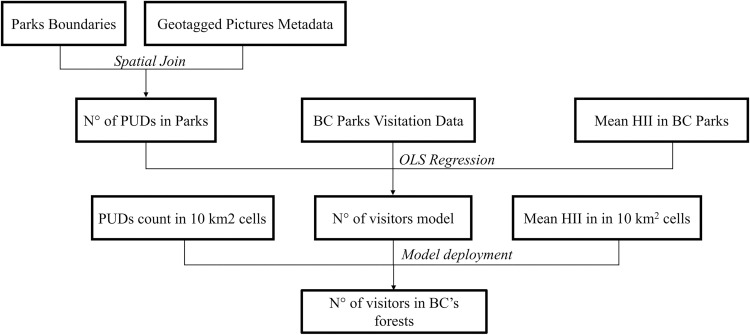
Outline of the method used for estimating the annual visitation rates in BC forests.

To estimate the number of annual visits in BC forests from crowdsourced social media data, we applied a similar methodology to the one applied by Ghermandi [39] and Sinclair et al. [40], who estimated the number of visitors in natural systems worldwide and wetlands in Kerala (India), respectively. However, while in these studies the authors used univariate models that only considered PUDs to estimate the number of yearly visitors, we included a second variable, the Global Human Influence Index (GHII). The decision to include GHII in our model was to account for the fact that the number of social media pictures acquired in the landscape is influenced by its accessibility [41]. As shown in Fig 2, we estimated the statistical relationship between the dependent variable (number of annual visits) and independent variables: (i) number of annual PUDs and, (ii) global human influence index (GHII) in a calibration sample. Then, we applied the estimated relationship to predict the number of forest visits outside the calibration sample. Specifically, we developed a multivariate ordinary least squares (OLS) model (Eq 1), calibrated using visitation statistics of BC provincial parks (Fig 2). The rationale of using an OLS regression was that a previous study [39] that compared the performances of various models in estimating the yearly visitation from PUDs determined that forward OLS regression yielded the best results together with the standardized major axis regression. Furthermore, OLS regression was used in [40] where the authors estimated the number of yearly visitors from PUDs, after calibrating their model in the context of the study region.

Visitation statistics were empirically collected (from 2007 to 2018) by BC Parks and made publicly available in an annual statistics report that contained annual attendance data for each provincial park in BC. In our analysis we used exclusively forest-dominated parks (forest cover ≥ 50%) to ensure consistency between the calibration sample and the areas in which we applied the OLS model. Therefore, the calibration sample included all the forest-dominated provincial parks in which BC Parks had published annual visitation data from 2007 to 2018 (N = 100), and the OLS regression parameters were estimated using PUDs detected in the parks, within the same time window. GHII value for BC provincial parks were estimated averaging the GHII values of each cell that fell into the park boundaries. To this end the zonal statistic ArcGIS pro tool was used. Since the dependent variable cannot be negative, we performed a logarithmic transformation on both the dependent and independent variables. Following [39] the PUDs count in each park is increased by one to avoid attempts to calculate the logarithm of zero in parks in which no geotagged pictures were detected.The OLS model had the following functional form:

$$\log\left(y_{i}\right)=\beta_{0}+\beta_{1}\log\left(x_{i}\right)+\beta_{2}\log(x_{ii})+\epsilon_{i}$$ Eq 1

where y_i_ is the average annual attendance for the i^th^ provincial park, and x_i_ is the average annual number of PUDs and x_ii_ is the average GHII value for the provincial park. Model coefficients were estimated using the Python package Statsmodels. We tested the assumption of normality for the distribution of the residuals with the Shapiro-Wilk test and the hypothesis of covariance between the two independent variables. To predict the number of visits from the log-transformed outputs, we used both the naïve forecast technique (exponential transformation of the logarithmic predictions) and the Duan’s Smearing technique [42]. The visit estimates obtained with these two approaches were then compared using the actual parks visitation data to determine which one yielded the most accurate results, using the Root Mean Square Error (RMSE) metric. Using the same metric, the performance of the multivariate OLS regression model was compared with a traditional univariate model (calibrated on the same dataset). This allowed us to determine if the inclusion of GHII resulted in an improvement of the model accuracy. Lastly, we applied the OLS model to estimate the number of annual visits in each 10 km^2^ cell of a grid covering the entire province. The GHII values in each cell were estimated using the zonal statistics tool of ArcGIS pro.

#### Value of forest recreational sites

**Fig 3:**
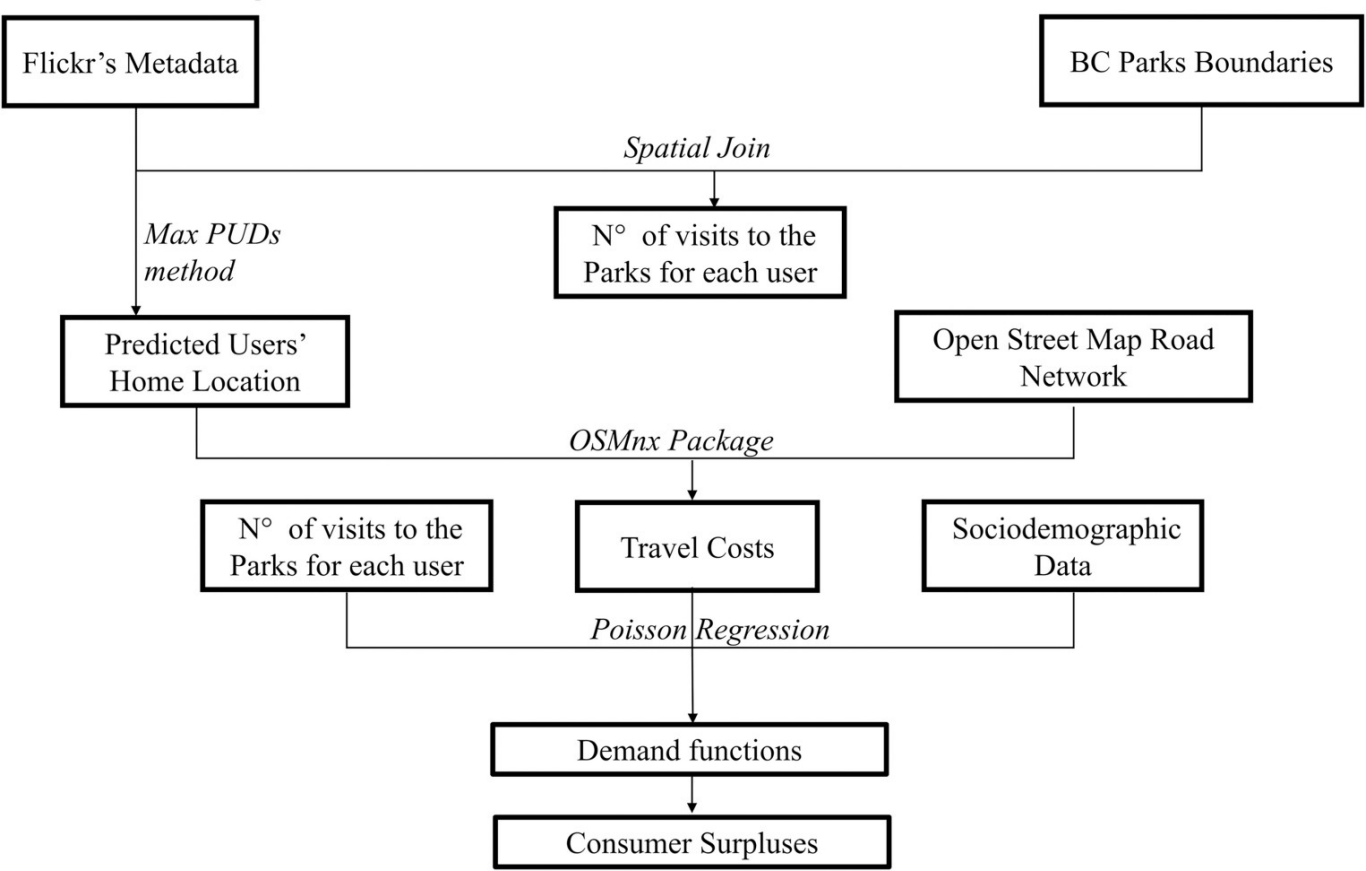
Outline of the methodology used for estimating the monetary value of the recreational ecosystem service provided by BC forested provincial parks.

To assess the monetary value associated with the recreational ecosystem service provided by BC forests, we applied the single-site individual travel cost method using crowdsourced social media data (Fig 3). The travel cost method (TCM) from crowdsourced social media data has been applied in the past by Ghermandi [43] and Sinclair et al. [21]. We applied the method only in forest dominated (forest cover ≥ 50%) Provincial Parks. Demand functions (Eqs 2 and 3) were estimated using a negative binomial regression, with the number of visits made by each detected visitor as the dependent variable and the following independent variables: (i) total travel cost to the site, (ii) visitor’s income, (iii) travel cost to the nearest substitute site. In an effort to reduce the incidence of multi-purpose and multi-day trips, which can bias the results of TCM analyses [13], we introduced four criteria that each PUD had to respect to be considered a visit: (i) the author must be a domestic visitor according to the max-PUD methods results, (ii) the distance traveled by the author for the round trip to the site must not exceed 400 km, (iii) the author must not have acquired pictures of the same provincial park for the following seven days, and, (iv) more than 50% of the pictures constituting the PUDs must have been acquired within provincial park boundaries. The total travel cost includes two components: (i) the costs of a round trip to the site, and (ii) the opportunity cost of the time spent traveling. To estimate the cost of traveling to the site, we assessed the distance traveled by each visitor using the Python package OSMnx. This package allows the user to download geospatial data from OpenStreetMap, and then model the drivable urban networks. The travel distance was estimated as the most efficient route between the estimated visitor home location and the coordinates of the PUD. To obtain the cost of traveling, the estimated travel distance was then multiplied by the average cost of driving a private car, retrieved from the Canadian Automobile Association [32]. Lastly, the opportunity cost of the time spent traveling was calculated as 1/3 of the estimated hourly income rate derived from BC census data (Table 1), as is commonly done in travel cost analysis [44]. We assigned to each park an alternative provincial park as the park closest to the visitor estimated home location. The travel cost for a round trip to the substitute site was then estimated applying the same travel cost estimation.

Once all the variables were obtained, we excluded all provincial parks with less than 40 detected visitors to ensure that our sample size respected a minimum threshold of 10 users per variable as suggested by Ghermandi [39]. The functional form of the estimated demand function was the following.


$$\log\left(y_{i}\right)=\beta_{0}+\beta_{TC}x_{TCi}+\beta_{I}x_{Ii}+\beta_{s}x_{si}+\epsilon_{i}$$ Eq 2

Where y is the number of PUDs by visitor i^th^ for the recreational site considered, β0 is the intercept of the model, x_TCi_ is the cost for a round trip to the recreational site considered for visitor i^th^, x_Ii_ is the individual income of the visitor i^th^, and x_si_ is the travel cost for a round trip to the substitute site for visitor i^th^. Lastly, the consumer surplus generated by a visit to the site was estimated following Creel and Loomis [45]:

$${CS}_{visit}=-\frac{1}{\beta_{TC}}$$ Eq 3

Where *CS*_*visit*_ is the consumer surplus generated by each visit to a site and *β*_*TC*_ is the coefficient of the travel cost variable in the model.

## Results

We gathered the metadata of 1,719,130 geotagged pictures *via* Flickr API. Only 365,477 of these pictures (21.26%) were acquired in forested areas and therefore retained for further analysis. These pictures were uploaded by 12,532 Flickr users and resulted in 44,102 PUDs.

### Visitors home location and visitation trends

**Fig 4:**
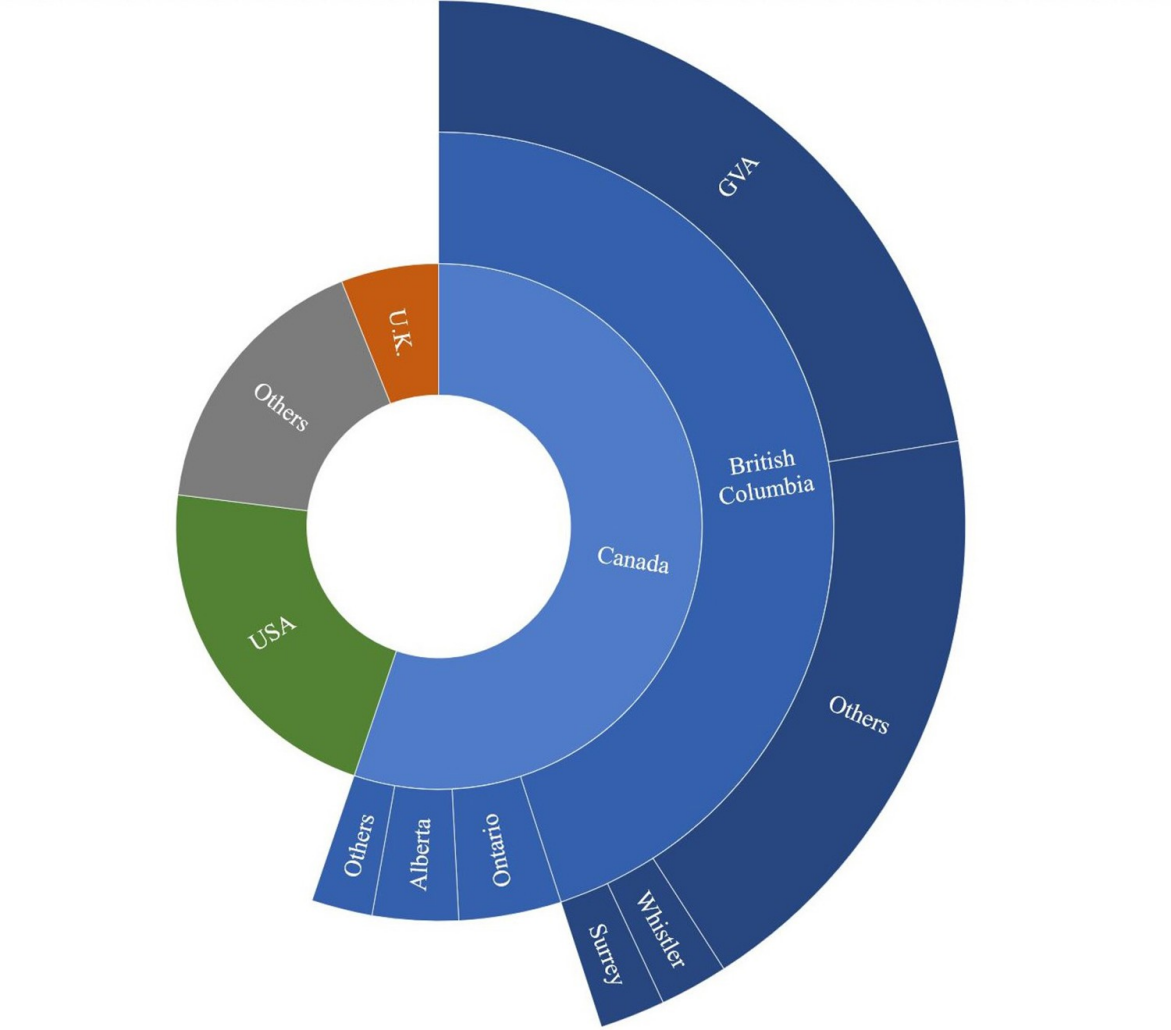
Place of origin of Flickr users that visited BC forests. Deduced from the maximum PUDs method. GVA = Greater Vancouver Area.

Among the 12,532 Flickr users that acquired at least one picture between 2005 and 2020, 10,399 satisfied the 10 pictures requirement needed to apply the max PUDs methodology and were included in the visitor’s home location analysis. We compared the results of the max PUDs method with the country of origin indicated by the authors in their public profiles and found a 84% correspondence. The majority of the users are Canadian (55.2%). International visitors come from 92 countries, the 5 main countries being (in declining order): United States (48.5%), United Kingdom (13.3%), Australia (5.0%), Germany (3.9%) and Netherlands (2.5%) (Fig 4). Applying the max PUDs method on Canadian users, we found that most came from BC (81.6%) and Ontario (7.6%). Finally, among the visitors from BC, the most frequent districts of origin are: Greater Vancouver Area (22.4%), Whistler (4.1%) and Surrey (4.0%).

**Fig 5:**
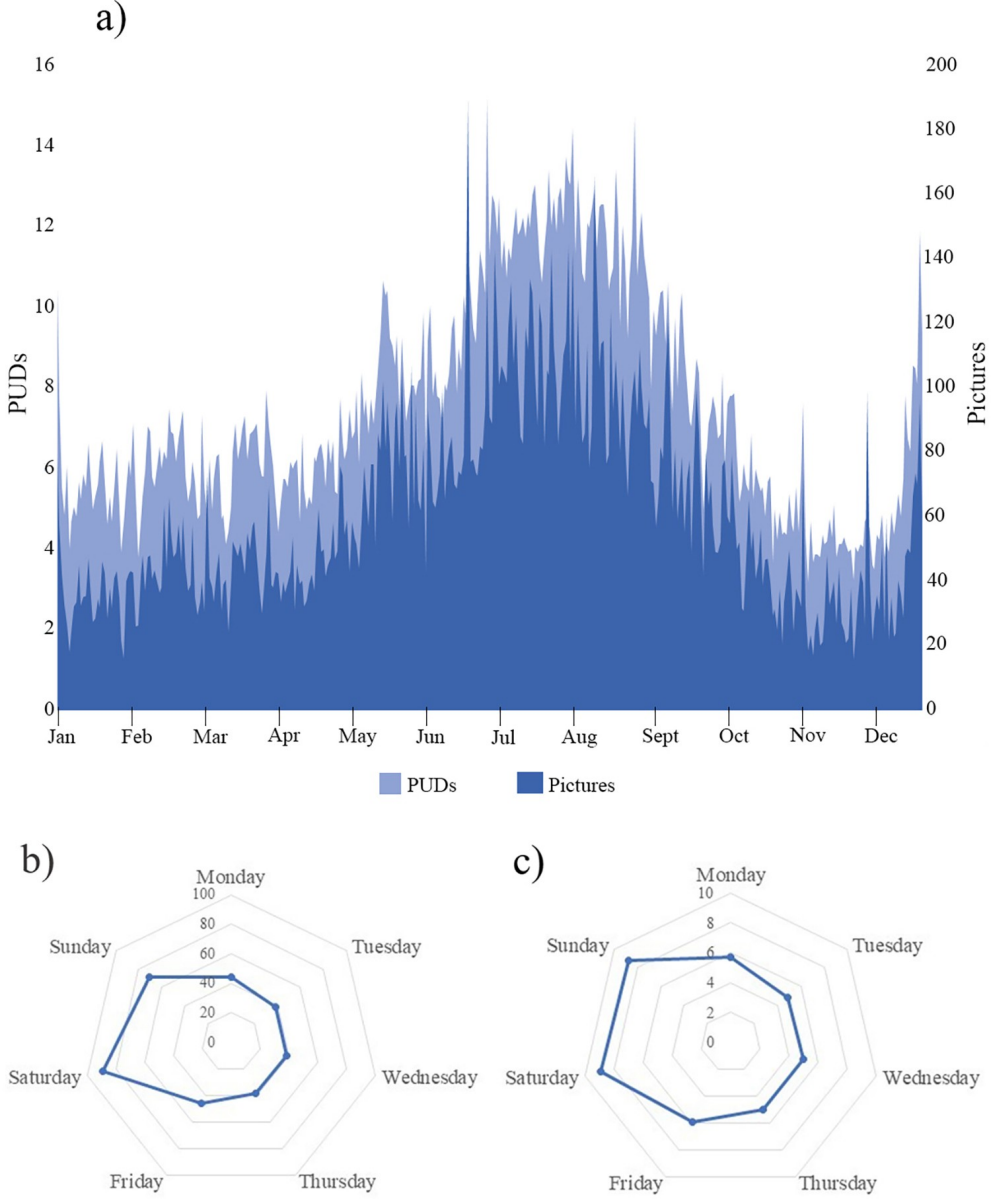
Temporal trends of forest recreation consumption in BC. Values are averages obtained grouping the timestamps of the entire dataset of relevant pictures: (a) monthly variations in numbers of pictures (dark blue) and PUDs (light blue), and average number of pictures (b) and PUDs (c) over weekdays.

From the timestamps of all 365,477 gathered pictures acquired in forested areas we inferred the temporal pattern of forest recreation consumption in BC. As shown in Fig 5A, consumption of forest recreation peaked during the summer months and the end of December, while it was lowest in October and November. As expected, the consumption of forest recreation furthermore peaks during the weekend while remaining almost constant during the working week (Fig 5B and 5C).

**Fig 6:**
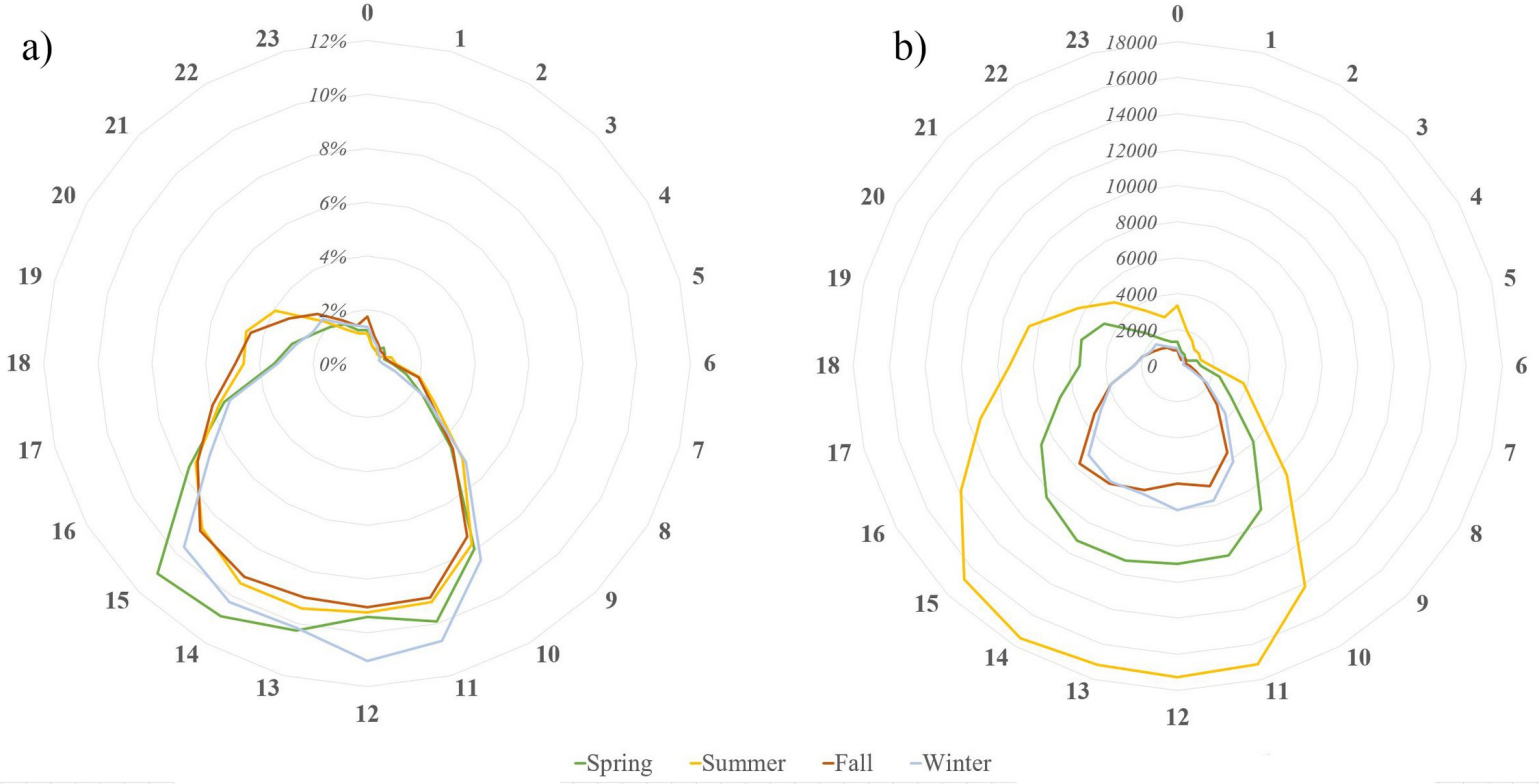
Temporal (hourly) trends of picture acquisition in BC forests, across the seasons: a) total number of pictures, b) percentage of pictures (over the total of the season). Time of acquisition 0 includes pictures acquired between midnight and 12:59 AM local time, 1 includes pictures acquired between 1:00 and 1:59 AM, etc.

We further used the timestamps to explore the hour of the day during which the pictures were acquired across the four seasons. As shown in Fig 6A (and in accordance with Fig 5A), most pictures were taken in summer months, followed by spring, winter, and fall. As expected, the majority of pictures were acquired during daylight between 10:00h and 15:59h, irrespective of the season. Fig 6B shows how the pictures acquired in each season are distributed during the day. Summer and fall months have similar picture distributions, while winter months have a distinctive peak between 11:00h and 12:59h and spring months between 14:00h and 15:59h.

**Fig 7:**
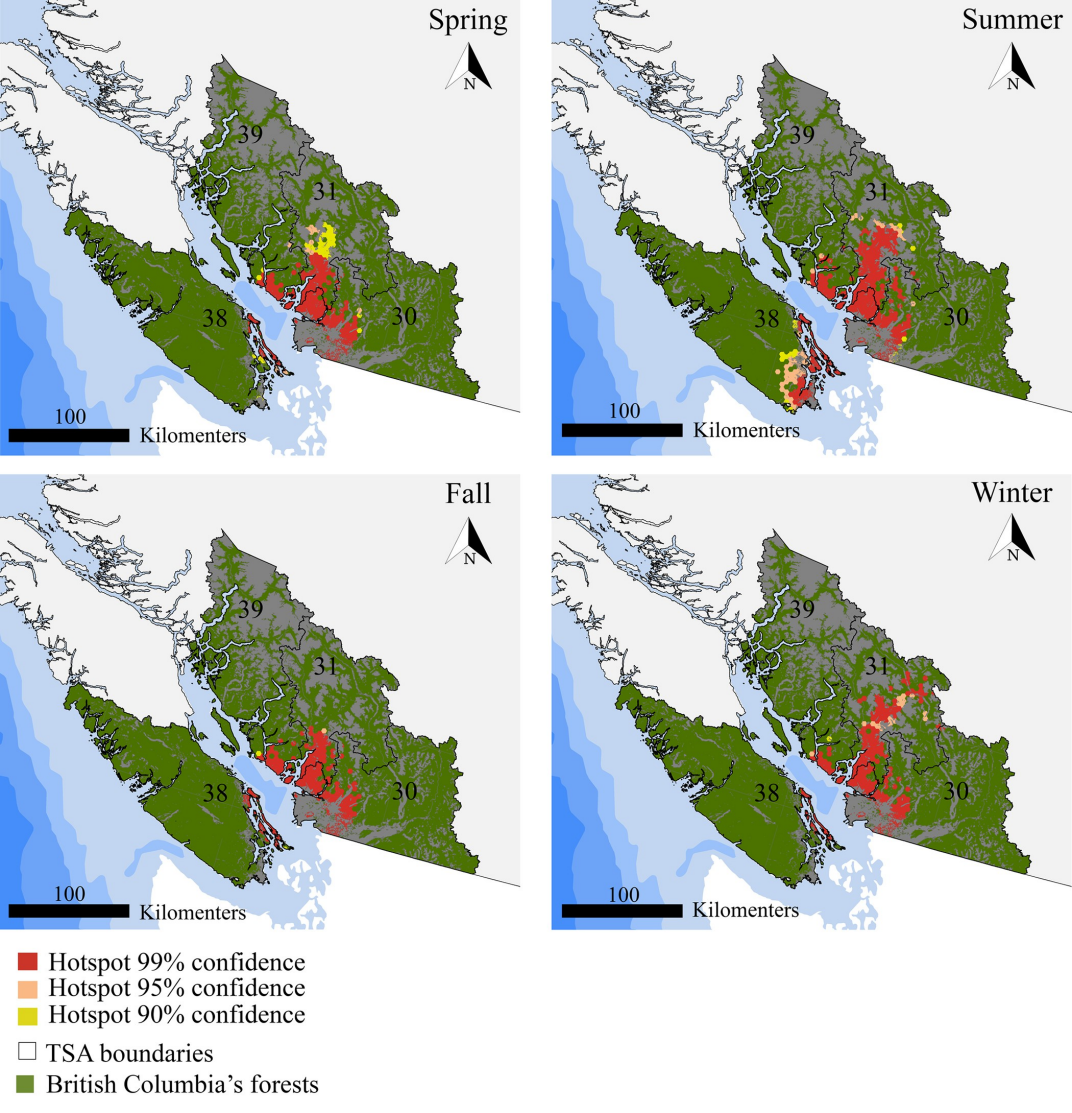
Seasonal hotspots of forest recreation in south-western BC. The confidence value indicates the probability of the cell being a hotspot according to the Getis-Ord Gi* statistic [38]. The size of the cells is 5.4 km^2^. Geodetic datum: NAD 83. Source of layers Natural Earth (https://www.naturalearthdata.com/).

Lastly, combining picture locations and dates, we identified hotspots of recreation consumption. Throughout the year, hotspots of forest recreation are located in the south-west of BC, specifically in TSAs 30, 31, 39 and 38 (Fig 7). Hotspots during spring and fall are more spatially constrained. However, spring hotspots expand further north into TSA 31. Summer and winter hotspots are distributed differently: Summer hotspots expand further into the inland of southern Vancouver Island (TSA 38) and winter hotspots expand further into the forests north of Whistler (TSA 31). Recreational hotspots in the south of TSA 39 and surroundings are present throughout the year.

### Number of annual visits in British Columbia’s forests

Of the 1,719,130 geotagged pictures gathered in BC, 19,176 were acquired within the boundaries of a forested provincial park by 1,641 unique visitors who, on average, visited the parks 2.38 times and took 4.91 pictures on each visit, resulting in a total of 3,908 PUDs. GHII ranges from 0 (low impacts) to 65 (high impacts) and the mean GHII value for BC provincial parks is 16.4 with Hemer park being the one with highest GHII (46.0), and Purcell wilderness conservancy park being the one with the lowest GHII (1.3). Table 2 presents the coefficients of the two OLS linear regression models calibrated on the provincial parks’ visitation data. As shown, both models highlight a statistically significant relationship between the number of visitors reported by BC Parks statistics and the number of detected PUDs (p-value < 0.001). Furthermore, in the bivariate model the mean GHII value is also statistically significant (p-value < 0.001). Table 3 presents the performance of the two models and shows that model that include both the number of PUDs and the average GHII of the park (bivariate OLS) outperform the univariate OLS both in terms of variation of the dependent variable explained (R-squared) and root mean square error. Lastly, comparing the performances of the naïve transformation and Duan’s smearing technique, results show that the smearing transformation is the best suited to transform the log predictions of the model to the actual number of annual visits.

**Table 2:** Results for the univariate and bivariate OLS log-log regression models, in parenthesis we report standard errors.

Coefficient	Univariate OLS	Bivariate OLS
Intercept ***	9.2086 (0.145)	7.8389 (0.357)
Slope PUDs***	1.1532 (0.091)	1.0628 (0.096)
Slope GHII***	-	0.5642 (0.138)
Akaike Information Criterion	327.6	274.3

Note

*, **, *** denote significance at the 10%, 5%, and 1% level, respectively, in parenthesis we report standard errors.

**Table 3:** Performances of the univariate and bivariate OLS log-log regression models.

Parameter	Univariate OLS	Bivariate OLS
R-squared:	0.537	0.654
Root Mean Square Error Naïve	127,194	106,607
Root Mean Square Error Smearing	118,731	101,922

The Shapiro-Wilk test did not reject the hypothesis of normal distribution of the residuals neither for the univariate nor for the bivariate model, also in the bivariate model the two independent variables do not present multicollinearity.

**Fig 8:**
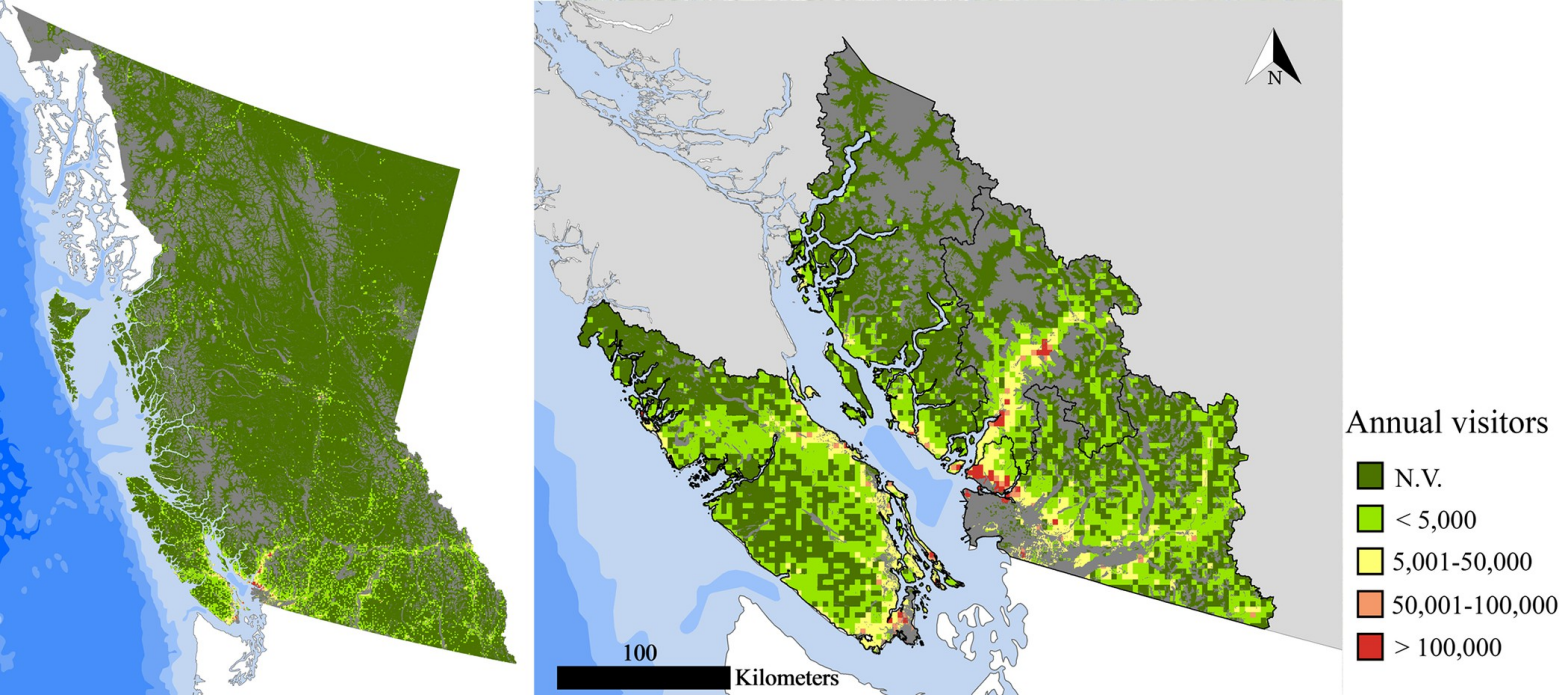
Estimated number of annual visits in BC forests (left) and south-western BC (right). Values are average annual number of visits between 2005 and 2020. Geodetic datum: NAD 83. Source of layers Natural Earth (https://www.naturalearthdata.com/).

We then estimated the visitation rates of BC forests for the whole province (Fig 8), applying the OLS regression model to the count of PUDs and the mean GHII value inside each 10 km^2^ cell covering the province and implementing the naïve transformation to the model predictions. For this analysis all the 44,102 PUDs detected in BC forests from 2005 to 2020 were considered. The total number of recreational experiences (by both national international visitors) from 2005 to 2020, was on average > 44.2 million per year. As expected, the geographic distribution of these recreational experiences is not homogeneous across the province. Over 54% of all recreational visits to BC forest are concentrated in TSA 30 (surrounding Greater Vancouver) and TSA 38 (surrounding Greater Victoria).

### Value of forest recreational sites

The 3,908 PUDs detected in forest-dominated BC provincial parks resulted in 2,814 visits, made by 962 visitors. As shown in Table 4 the average income of these visitors is 71,237 CAN$/year, the average travel cost incurred by the visitors is CAN$ 48.5 while the average travel cost to the substitute site is CAN$ 22.6.

**Table 4:** Summary statistics for the data used in the negative binomial regressions.

Variable	Average	Range	Std. Dev.
Income (CAN$)	71,237	33,380–123,648	12,848.33
Travel Cost to provincial park (CAN$)	48	1–168	38.63
Travel Cost to substitute provincial park (CAN$)	23	1–322	21.98

**Fig 9:**
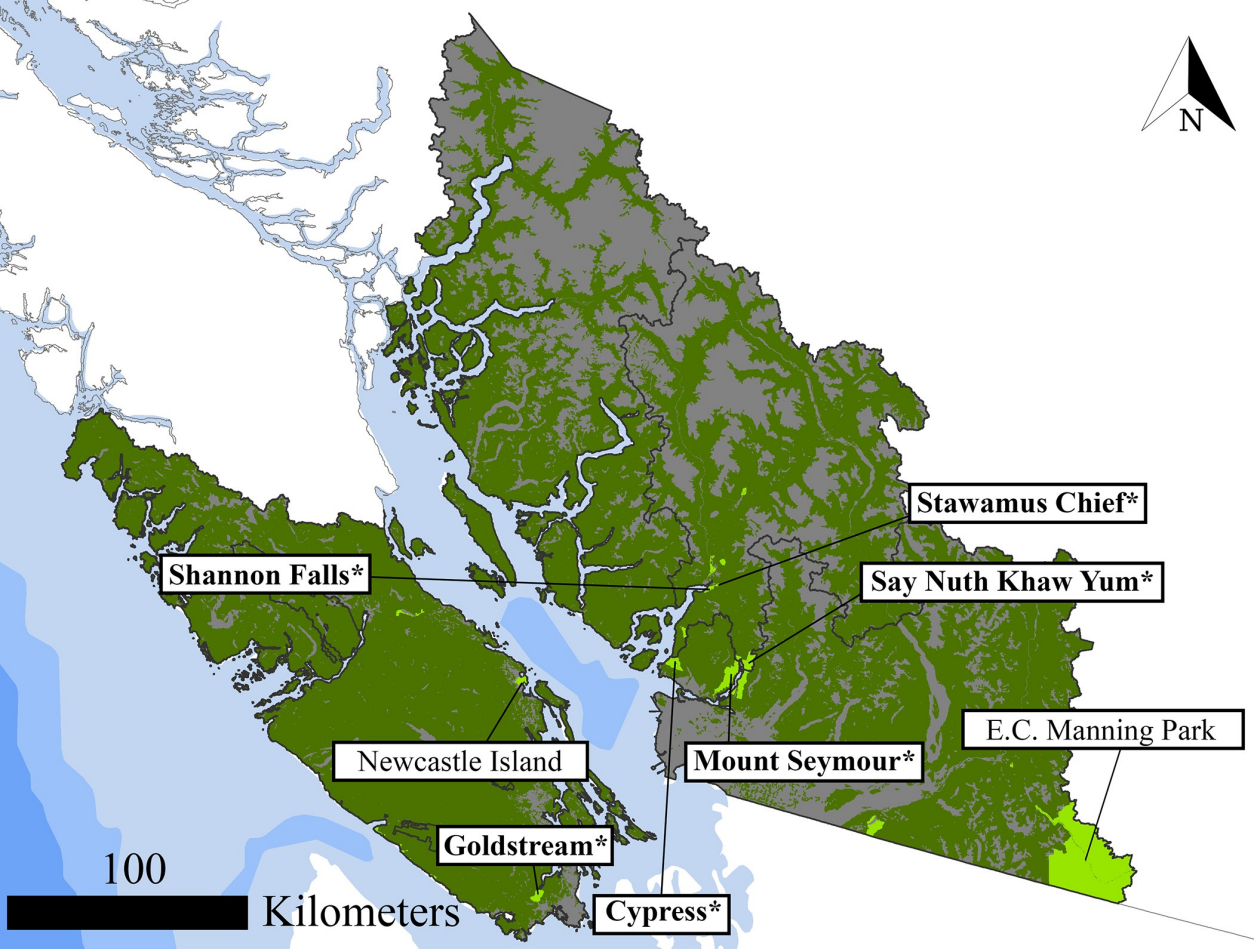
Locations of the forested provincial parks in which we administered the negative binomial regression. * denotes the parks in which the regressions yielded statistically significant results. Geodetic datum: NAD 83. Source of layers Natural Earth (https://www.naturalearthdata.com/).

In total, 8 forested provincial parks in BC had sufficient numbers of detected visitors (*i*.*e*., at least 10 visitors per predictor variable in the model) to apply the crowdsourced travel cost method (Tables 2 and 3). In each negative binomial regression, the coefficient of the travel cost variable (TC) has the expected negative sign, meaning that the number of visits made by a visitor to a site decreases when the cost of traveling to the site increases. However, the negative binomial regressions yielded statistically significant results only for 6 of 8 forested provincial parks initially included in the analysis (Table 5 and Fig 9). As noted in Table 5, all significant results can be found in parks where the number of detected total visitors exceeds 50. The other variables that we included in the model, income (Inc) and travel cost to the nearest substitute site (TC sub. site), have poorer performances, statistically significant in only three and two provincial parks respectively. However, the estimated coefficients had the expected positive signs indicating that the number of visits made to a site is expected to increase for visitors with higher income and when the cost of traveling to the closest substitute site increases. As expected, the performance of the negative binomial regression, measured with the Akaike Information Criterion (AIC), generally increases as the number of detected visitors increases. As can be seen in Fig 9, all the forested provincial parks in which we estimated statistically significant coefficients are located near the largest urban centers of BC, *i*.*e*., Greater Vancouver Area and Greater Victoria. The only forested provincial park in which more than 50 visitors were detected that is not statistically significant is E.C. Manning Provincial Park, which is situated more than 150 km east of Vancouver. The average consumer surplus estimated with the Creel and Loomis approach ranges from CAN$ 29 estimated in Mount Seymour Provincial Park and Say Nuth Khaw Yum (Indian Arm) Provincial Park, to CAN$ 87 estimated in Goldstream Provincial Park.

**Table 5:** Results of negative binomial regression for the detected visitors in BC forested provincial parks.

Park Name	dF	AIC	Int.	TC	Inc.	TC sub. site	CS (CAN$)	Tot. CS (M CAN$)
Stawamus Chief	197	986.4	1.1410 (0.873)	-0.0140*** (0.005)	0.002 (0.001)	-0.0087 (0.006)	71	21.286
Mount Seymour	129	896.4	1.1887*** (0.419)	-0.0328*** (0.005)	0.0301*** (0.007)	0.0084* (0.005)	30	19.379
Cypress	152	963.4	0.9765*** (0.308)	-0.028* (0.004)	0.0195** (0.007)	-0.0045 (0.005)	36	34.913
E.C. Manning	101	546.9	1.9166 (0.349)	-0.0011 (0.002)	-0.0188 (0.01)	-0.0075 (0.004)	N.S.	N.S.
Shannon Falls	84	301.7	1.2895 (0.877)	-0.013* (0.005)	-0.0033 (0.018)	-0.00114 (0.01)	77	27.150
Goldstream	53	230.15	-1.4021** (0.596)	-0.0115*** (0.003)	-0.0607*** (0.014)	0.0028* (0.002)	87	29.750
Say Nuth Khaw Yum	52	250.1	5.2387 (0.908)	-0.034* (0.015)	-0.1012 (0.019)	0.015 (0.02)	29	2.908
Newcastle Island	38	178.9	1.0695 (0.927)	-0.0058 (0.005)	-0.0096 (0.024)	0.0095 (0.011)	N.S.	N.S.

dF = degree of freedom, AIC = Akaike information criterion, Int. = Intercept, TC = travel cost, Inc. = income, TC sub. Site = Travel cost to substitute site, CS = Average consumer surplus, Tot. CS = total Consumer Surplus in millions of CAN$

Note

*, **, *** denote significance at the 10%, 5%, and 1% level, respectively, in parenthesis we report standard errors

Combining the estimates of the annual number of visits made by domestic visitors (obtained *via* the OLS linear regression) and the average consumer surplus per visit obtained with the negative binomial regression, it was possible to estimate the total annual consumer surplus of the seven parks, providing insights into the monetary value of the recreational ecosystems service provided by the park per year. The provincial park with the highest total annual consumer surplus is Cypress Provincial Park (~ CAN$ 34 million).

## Discussion and conclusion

Crowdsourced social media data offers valuable insights into the cultural services provided by ecosystems. The high volume, low-cost, fast data collection, and large scale applicability of social media data [16] provide new opportunities to study the demand for forest recreation, having the potential to drastically reduce the time and costs associated with on-site surveys in remote areas. Thus, their application in the study of nature-based recreation is a fast-growing research field. The overarching objective of our research was to assess this potential, especially in tackling the mapping and valuing challenges that have limited the inclusion of recreational ecosystem services in forest management plans. Our analysis was structured on three levels. First, we characterized the consumption of forest recreation, identifying inter- and intra-annual trends, recreation hotspots and the place of origin of visitors. Second, we quantified the consumption of forest recreation with an OLS regression model, calibrated with empirically collected visitation data in BC provincial parks, and applied it to the entire province. Third, we performed a travel-cost analysis to estimate recreational values in BC Parks, relying on crowdsourced and secondary data. For each of these three levels of analysis, we will discuss our findings and their limitations, as well as the opportunities for forest management associated with crowdsourced social media data. Lastly, we will discuss future directions for the use of crowdsourced social media data to analyze forest recreation ecosystem service.

### Crowdsourced social media data to characterize forest recreation consumption

Using the timestamps of Flickr pictures we were able to identify the annual, weekly, and hourly trends in forest recreation consumption. Our findings show that the consumption of forest recreation peaks during the summer and during the weekends, in line with expectations and with the results of previous studies [24]. The hourly distribution of the pictures acquired in BC forests is also similar to the one described by Ciesielski et al. [27], with most of the pictures being acquired between 10:00h and 16:00h. Combining the geotags and the timestamps of the gathered pictures, we were able to identify and differentiate seasonal hotspots of forest recreation consumption. As expected, recreational hotspots are located in the southwest of the province, around the main urban centers: Vancouver and Victoria. The spatial distribution of the hotspots aligns with previous findings that the data volume obtained from crowdsourcing social media is higher in areas with large populations than in remote areas [19]. Information such as temporal and geographic trends of recreation consumption could help forest managers plan harvesting operations, so as to minimize impacts on the provision of recreational ecosystem service. However, the fact that remote areas tend to be underrepresented by crowdsourced social media data should be considered carefully, and an integration of crowdsourced social media data and traditional surveys methods could be a better option. Moreover, the pre-selection of pictures by social media users before posting content could introduce biases towards: (i) certain hours of the day (e.g., due to occurrence of sunrises and sunsets), (ii) days in which certain atmospheric phenomena occurs (e.g., rainbows, *aurora borealis*), and (iii) seasons characterized by certain phenological events (e.g., blooming and autumnal leaves color change). Lastly, the presence of wildlife, especially in the case of certain charismatic species such as grizzly bear (*Ursus arctos horribilis*) or wolf (*Canis lupus*), could also introduce biases, as already suggested by Tenkanen et al. [19].

Our results confirm the reliability of the max PUDs methodology in predicting the home locations of social-media-users as found by Sinclair et al. [24]. We obtained a slightly lower precision at the country level than Sinclair et al. [24] (84% with a 10 PUDs threshold *versus* 90%). Few Flickr users declared their home location to a finer level of spatial detail than country, making it impossible to assess the reliability of the method for home cities or sub-national regions. Comparing the predictions of the max PUDs methodology with the data on international visitors’ arrivals from Statistics Canada [46], it appears that crowdsourced data from Flickr performed well in predicting the country of origin for Europeans, Americans and Australians, while systematically underestimating the number of visitors from Asia. In fact, while Asian visitors constitute on average over 16% of the international visitors of BC, they have acquired only 3% of the pictures gathered in this study (according to the results of the max PUDs methodology). This discrepancy could be explained with different accessibility of online photo-sharing services across different countries, in particular China [39].

### Crowdsourced social media data as a proxy for forest visitation rates

We found a strong correlation between the number of PUDs detected, the GHII value and the number of recreationists visiting forest dominated provincial parks. Various studies estimated univariate models in the past, using exclusively PUDs as the independent variable. Wood et al. [17] observed correlations at 839 worldwide recreational sites (r^2^ = 0.39), Keeler et al. [20] for North-American lakes (r^2^ = 0.65 and 0.70) and Sinclair et al. [40] for the Kerala’s wetland ecosystems network (India) (r^2^ = 0.63). In our study, the provincial parks that we used to calibrate our model range from highly frequented peri-urban areas such as Cypress Provincial Park (averaging over 1,200,000 annual visits observed between 2007 and 2018) to remote areas far from any major urban centers such as the Kinaskan Lake Provincial Park (averaging less than 15,000 annual visits observed between 2007 and 2018). Despite this wide range of empirically detected visits, the performances of our calibration model (r^2^ = 0.65) lie in the upper limit of the above-mentioned models. The statistical significance of the GHII indicator in our model aligns with the notion that the number of geotagged pictures acquired in a landscape is influenced by its accessibility and closeness to urban areas [26, 41]. Comparing the performances of the bivariate model (with both PUDs and average GHII as independent variables) with the performances of a traditional univariate model (with only PUDs as independent variable) across our study area we found that the bivariate model outperforms the univariate model. This indicates that GHII is a potentially a useful indicator that could easily be integrated in future models attempting to estimate the number of visitors in the natural landscape from crowdsourced social media data.

We recognize that, when applying the OLS model outside its calibration sample (*i*.*e*., provincial parks) to estimate the number of recreationists in forested areas in the whole province, the results are potentially subject to biases. Most notably, social media users may not be a representative sample of the entire population of recreationists, as they are usually younger [47], and different social media draw the interest of different sections of the general population [16]. However, empirical studies [48] indicate that Flickr is less biased towards younger people that Instagram and Twitter. Also, previous studies have shown that this limitation can be partially mitigated by crowdsourcing data from different social media [19]. Another limitation of crowdsourced social media data is the fact that not every recreational activity traditionally carried-out in forested areas is equally compatible with the acquisition of pictures to be shared on social media. In fact, among the main recreational activities in which forest visitors engage, as listed by Rosenberger et al. [49], are activities such as hunting and motorized boating, which appear to be less likely to be portrayed in pictures than hiking or camping. Therefore, relying exclusively on crowdsourced social media data to estimate the number of visits in forested areas could result in failing to recognize important recreational areas devoted to specific forest recreational activities, or areas that are mostly used by a certain segment of the general population. Despite these limitations, crowdsourced social media data offer unparalleled opportunities to estimate forest visitation rates at large-scale with relatively high spatial resolution. Visitation rates maps—such as the ones that we were able to generate in our study—could provide a useful benchmark to forest managers. For example, by overlaying a map of forest visitation rates with a map of timber values, it becomes possible to identify areas were the trade-off between timber production and forest recreation has to be carefully balanced. In conclusion, this innovative approach can produce valuable outputs that could help in tackling the issue of mapping nature-based recreation an effectively account for its values in forest management.

### Crowdsourced social media data for travel-cost analysis in forest sites

In our final analysis, we used crowdsourced geotagged pictures as a data source to perform a monetary assessment of the recreational values of BC forested provincial parks. The use of the OSMnx package has allowed us to simultaneously estimate the travel distances of over 900 visitors across the entire BC provincial parks system. Previous studies that adopted the crowdsourced travel cost methodology have instead used the Google Maps API [21, 50]. However, Google Maps API implements a freemium strategy, offering for free a defined number of requests (1000), limiting the extent of the analyses. On the contrary OSMnx enables the user to make an unlimited number of requests making our approach a more scalable and innovative solution. Our results (ranging from CAN$ 29 to CAN$ 87 of consumer surplus per visit) are consistent with the values estimated with traditional surveys for forest recreation in forests in the North-West of the United States of America, where the average consumer surplus of forest recreation is 67 US$ (83 CAN$) [49]. In their application of the crowdsourced travel cost method to a set of wetlands, Ghermandi [50] obtained significant results despite having fewer available data (10 visitors per variable included in the regression), while we obtained significant results only in forested provincial parks where we detected more than 12 visitors per variable. The poor performance of the crowdsourced travel cost method in our study area could be explained by the geographical distribution of BC population. In fact, over 57% of BC inhabitants reside in either Greater Vancouver or Greater Victoria. The coastal location of both Vancouver and Victoria is likely to introduce a bias in the determination of the exact coordinates of the home locations of the authors because of the ocean truncation effect. This, combined with the fact that the precision of the maximum PUDs method drops when predicting the place of origin of an author at the municipality level (Sinclair et al., 2020), hampers the reliability of distances traveled in our dataset. As a result, the use of the travel cost method from crowdsourced social media data appears to be especially appropriate for peri-urban forests that are accessible on a single-day trip from multiple municipalities. When estimating the monetary value of the recreation ecosystem service provided by forest located further from urban areas, traditional survey methods (*i*.*e*., *in-situ* and mail) might be more suitable. Another limitation of the crowdsourced travel cost method is the use of census data to estimate the income of recreationists. In Canada, census data are collected by Statistics Canada every five years. During these time windows people can move and change their occupation. In our study (as well as in previous applications of the crowdsourced travel cost method), the analysis spans over multiple years, therefore the ability of these data to approximate the recreationists incomes progressively decreases for visits made prior to and later than the census year. As mentioned above, another bias that can affect these analyses is that social media users are not necessarily representative of the general population. The development of techniques to deal with this issue is hampered by the limited socio-demographic data that can be gathered when performing a crowdsourced analysis in respect to traditional surveys. Furthermore, despite the implementation of weighing systems, such as the use of PUDs, the crowdsourced travel cost analysis may still be biased towards very active users. Despite these limitations the use of crowdsourced data, combined with traditional non-market evaluation approaches (such as travel cost) and openly accessible data (such as Open Street Map) is an innovative and promising solution to the issue of valuing the recreational services provided by an ecosystem. Lastly, it is important to note that whenever crowdsourced data are used, ethical concerns about the social media users’ privacy arise.

### Advantages and limitations of crowdsourced data in the study of forest recreation

The analyses that we carried-out allow us to identify both the advantages and limitations of the use of crowdsourced data in characterizing, mapping and valuing forest recreation. Our conclusions are briefly summarized below:

- **Characterizing forest recreation:** crowdsourced social media data can provide valuable information to forest managers. Picture time-stamps can be used to explore hourly, daily, and seasonal trends of forest recreation, while picture coordinates can be used to identify the hotspots of recreation and the countries of origin of visitors. However, pictures shared on social media may not be representative of all of the pictures taken by forest visitors. As a result, the screening process we used to select the most relevant pictures may have introduced some bias towards certain times of day, atmospheric or light phenomena, phenological events, and/or the presence of certain charismatic species. Lastly, when using crowdsourced social media data to estimate the visitors country of origin, the different accessibility and popularities of social media platforms globally could also introduce biases.
- **Mapping forest recreation:** by exploring the relationship between PUDs, GHII and the empirically determined number of visitors to forest recreational sites, it is possible to calibrate regression models to estimate the number of visitors in forested areas based on crowdsourced data. Using this approach, forest managers could map and quantify the use of forest recreational ecosystem services. Furthermore, such an approach can be applied across large areas, at fine spatial resolution and in near real time. However, the results of these models should be interpreted as preliminary values that forest managers could use to include the recreational ecosystem service when planning forest operations at the strategic scale, recognizing that the estimates can be affected by biases. For example, pictures tend to be concentrated in forested areas near large population centers and in areas with well developed infrastructure, possibly causing an overestimation of the intensity of forest recreation consumption near cities and an underestimation in remote areas. Lastly, some forest recreational activities appear to be less easily depicted in pictures than others.
- **Valuing forest recreation:** the crowdsourced travel cost approach offers the possibility to obtain rapid and cheap estimates of the monetary value of forest recreational opportunities. The results obtained are consistent with the economic theory (down-sloping demand curves) and similar to the one obtained with traditional surveys. However, the applicability of the crowdsourced travel cost method may be limited to popular, peri-urban recreational sites for which it is possible to obtain statistically significant results. Conducting a crowdsourced travel analysis the time horizon can be easily extended to multiple years. This, on one hand, has the advantage of allowing the collection of more data. On the other hand, socio-demographic data acquired from secondary sources, such as the census, progressively loose their ability to represent visitors adequately. Lastly, the performance of the method used to identify the home location of the visitors (max PUDs), may became poorer when determining specific administrative areas of origin (rather than Country) of a visitor.

### Future perspectives

Analysing crowdsourced social media data represents a promising approach to include recreational values in forest management plans, nevertheless, this does not imply that these data could already completely replace traditional *in-situ* surveys. As discussed, the use of crowdsourced data may introduce a number of biases in the interpretation of the results. For example, the results of our research suggest that crowdsourced social media data can provide estimates of the visitation intensity in forested areas, where the use of empirical in-situ surveys is challenging and costly. However, future studies that test this hypothesis, by comparing the results of estimates based on crowdsourced social media data with empirically determined visitor numbers, are required. Moreover, new studies that explore the relationship between trends of forest recreation (inferred by crowdsourced social media data) and forest stand characteristics (such age, species, etc.) could provide insights on how the consumption of forest recreation is impacted by stand dynamics and forest management, and evolves over time and space. These studies could ultimately facilitate the integration of recreational ecosystem services into forest models and decision support systems. Additional studies that promote the integration of crowdsourced social media data and the evaluation of non-market ecosystem services are needed. Other traditional non-market evaluation techniques, such as the benefit transfer approach, could be successfully integrated with crowdsourced social media data. To this end, the content of crowdsourced social media images could provide valuable information on the recreational activities popular at specific locations. Having access to this information could potentially allow forest economists to conduct fine-grained application of the benefit-transfer approach, since the various recreational activities that can take place in forests generate different economic surpluses. We believe that, in the future, modern deep learning algorithms used for image classification tasks, such as convolutional neural networks, could play an important role in such studies.
